# Inflammatory Phenotypes Predict Changes in Arterial Stiffness Following Antiretroviral Therapy Initiation

**DOI:** 10.1093/cid/ciaa186

**Published:** 2020-07-02

**Authors:** Christine Kelly, Willard Tinago, Dagmar Alber, Patricia Hunter, Natasha Luckhurst, Jake Connolly, Francesca Arrigoni, Alejandro Garcia Abner, Ralph Kamngona, Irene Sheha, Mishek Chammudzi, Kondwani Jambo, Jane Mallewa, Alicja Rapala, Robert S Heyderman, Patrick W G Mallon, Henry Mwandumba, A Sarah Walker, Nigel Klein, Saye Khoo

**Affiliations:** 1 Centre for Experimental Pathogen Host Research (CEPHR), University College Dublin, Dublin, Ireland; 2 Malawi Liverpool Wellcome Trust Clinical Research Program, Blantyre, Malawi; 3 Institute of Translational Medicine, University of Liverpool, Liverpool, United Kingdom; 4 Institute of Child Health, University College London, London, United Kingdom; 5 Kingston University, London, United Kingdom; 6 College of Medicine, University of Malawi, Blantyre, Malawi; 7 Liverpool School of Tropical Medicine, Liverpool, United Kingdom; 8 Institute of Cardiovascular Science, University College London, London, United Kingdom; 9 9MRC Clinical Trials Unit, University College London, London, United Kingdom

**Keywords:** inflammatory phenotype, arterial stiffness, ub-Saharan Africa

## Abstract

**Background:**

Inflammation drives vascular dysfunction in HIV, but in low-income settings causes of inflammation are multiple, and include infectious and environmental factors. We hypothesized that patients with advanced immunosuppression could be stratified into inflammatory phenotypes that predicted changes in vascular dysfunction on ART.

**Methods:**

We recruited Malawian adults with CD4 <100 cells/μL 2 weeks after starting ART in the REALITY trial (NCT01825031). Carotid femoral pulse-wave velocity (cfPWV) measured arterial stiffness 2, 12, 24, and 42 weeks post–ART initiation. Plasma inflammation markers were measured by electrochemiluminescence at weeks 2 and 42. Hierarchical clustering on principal components identified inflammatory clusters.

**Results:**

211 participants with HIV grouped into 3 inflammatory clusters representing 51 (24%; cluster-1), 153 (73%; cluster-2), and 7 (3%; cluster-3) individuals. Cluster-1 showed markedly higher CD4 and CD8 T-cell expression of HLADR and PD-1 versus cluster-2 and cluster-3 (all P < .0001). Although small, cluster-3 had significantly higher levels of cytokines reflecting inflammation (IL-6, IFN-γ, IP-10, IL-1RA, IL-10), chemotaxis (IL-8), systemic and vascular inflammation (CRP, ICAM-1, VCAM-1), and SAA (all P < .001). In mixed-effects models, cfPWV changes over time were similar for cluster-2 versus cluster-1 (relative fold-change, 0.99; 95% CI, .86–1.14; P = .91), but greater in cluster-3 versus cluster-1 (relative fold-change, 1.45; 95% CI, 1.01–2.09; P = .045).

**Conclusions:**

Two inflammatory clusters were identified: one defined by high T-cell PD-1 expression and another by a hyperinflamed profile and increases in cfPWV on ART. Further clinical characterization of inflammatory phenotypes could help target vascular dysfunction interventions to those at highest risk.

**Clinical Trials Network:**

NCT01825031.

## Teaser

Adults starting Antiretroviral Therapy (ART) with advanced immunosuppression can be phenotyped into 3 inflammatory clusters. One cluster was defined by high T-cell PD-1 expression and another by a hyperinflamed biological profile. Changes in arterial stiffness on ART varied according to inflammatory phenotype.

Chronic inflammation persists in people living with human immunodeficiency virus (PLWH) despite long-term suppressive antiretroviral therapy (ART), contributing to vascular dysfunction and noncommunicable diseases [1–3]. Low-income sub-Saharan Africa (SSA) faces an epidemiological transition in cardiovascular disease, as urbanization increases traditional cardiovascular risk factors [4, 5]. Hypertension is already highly prevalent; diabetes, dyslipidemia, and obesity are increasing [6–8]. Already, 80% of hemorrhagic strokes in people under 65 years occur in low- and middle-income countries [9]. Continued increases in noncommunicable disease in low-income SSA are of major public health concern and the focus of Sustainable Development Goal 3.4 [9, 10]. Life expectancy is increasing among PLWH in the region, and the effects of persistent chronic inflammation superimposed upon an evolving background of cardiovascular risk in an aging cohort are unknown [11, 12].

In high-income settings, drivers such as microbial translocation, persistent human immunodeficiency virus (HIV) replication and latent coinfections have been implicated in chronic inflammation [13–15]. Precise pathways remain unclear, with some individuals being affected more than others, and become even more complex in low-income settings where additional drivers of inflammation are multiple. Recurrent acute or subacute infections such as tuberculosis (TB), CMV, malaria, helminths, and invasive bacterial disease underpin an exhausted immune system [16–18]. Cotrimoxazole decreases immune activation, suggesting that modifying infection-related drivers may help reduce chronic inflammation [19]. Late presentation with low CD4 is still common in SSA and is associated with lymph node fibrosis and poor immune reconstitution on ART [20–22]. Immune activation has also been linked to poor ART adherence [23], food insecurity [24], and host genetics [25] in SSA cohorts.

A pragmatic approach to this complex process might be to identify common inflammatory phenotypes that identify those at highest risk from the complications of chronic inflammation, and hence target limited resources for risk reduction. This approach has been used successfully to guide treatment in other diseases characterized by chronic inflammation, such as asthma and systemic lupus erythamatosus (SLE) [26, 27].

Arterial stiffness has been used as a measure of vascular dysfunction in HIV studies as well as in other chronic inflammatory conditions [28–33]. Carotid femoral pulse-wave velocity (cfPWV) is a gold-standard measurement of arterial stiffness and has been validated as a physiological biomarker of cardiovascular events and mortality [29, 30]. We have previously shown that arterial stiffness, as measured by cfPWV, is increased in PLWH during the first 3 months of ART and in people experiencing unstructured treatment interruptions [3, 15].

We aimed to investigate whether PLWH starting ART with advanced immune suppression could be stratified into inflammatory phenotypes, and whether these phenotypes predicted changes in arterial stiffness following ART initiation.

## METHODS

Antiretroviral therapy–naive adults with a new HIV diagnosis and CD4 less than 100 cells/μL were recruited prospectively from the ART clinic and voluntary HIV testing clinic at Queen Elizabeth Central Hospital, Blantyre, Malawi, along with adults negative for HIV with no evidence of infection within the previous 3 months (full details of recruitment in [3]; most patients positive for HIV were recruited from the REALITY trial [NCT01825031]). In brief, participants underwent a detailed clinical assessment and blood draw for plasma storage at 2 (enrollment) and 42 weeks post–ART initiation. The enrollment visit for participants with HIV was 2 weeks after ART initiation to minimize visit burden in this unwell group. cfPWV was assessed on all participants at enrollment and 10, 22, and 40 weeks later. All participants provided informed written consent and ethical approval was granted by the College of Medicine Research and Ethics Committee (COMREC), University of Malawi (P.09/13/1464), and the University of Liverpool Research and Ethics Committee (UoL000996).

Surface immunophenotyping of T cells was performed on fresh peripheral blood mononuclear cells using flow cytometry as previously described [3]. T-cell activation, exhaustion, and senescence were defined as CD38/HLADR, PD-1, and CD57 expression, respectively. Monocytes were defined as classical (CD14^++^CD16^−^), intermediate (CD14^++^CD16^+^), or nonclassical (CD14^+^CD16^+^). Stored plasma was tested for 22 cytokines: Proinflammatory Panel 1 (interferon [IFN]-γ, interleukin [IL]-1β, IL-2, IL-4, IL-6, IL-8, IL-10, IL-12p70, IL-13, tumor necrosis factor α [TNF-α]), Vascular Injury Panel 2 (serum amyloid A [SAA], C-reactive protein [CRP], vascular cell adhesion molecule 1 [VCAM-1], intracellular adhesion molecule 1 [ICAM-1]), Chemokine Panel 1 (macrophage inflammatory protein [MIP-1β], interferon gamma-induced protein [IP-10], monocyte chemoattractant protein [MCP-1]), Angiogenesis Panel 1 (vascular endothelial growth factor [VEGF-A], basic fibroblastic growth factor [bFGF]), and single analyte assays for IL-1R antagonist and IL-7, all from Meso Scale Discovery (MSD, Rockland, MD). Assays were performed following the manufacturer’s instructions and recommended dilutions for human plasma. Soluble CD163 was measured in plasma diluted at 1:20 using DuoSet antibodies (R&D Systems, Minneapolis, MN) on MSD Multiarray plates. cytomegalovirus (CMV) viral loads were quantified by DNA polymerase chain reaction (PCR) in a subset of participants with available plasma as described previously [34]. A negative CMV PCR was defined as less than 100 copies/mL and corresponded with values less than 3 cycle thresholds above background fluorescence.

### Statistical Analysis

Categorical and continuous variables were compared using chi-square and Kruskal-Wallis or Wilcoxon rank-sum tests, respectively. We used principal component analysis (PCA) to reduce the dimensionality and to eliminate redundancy across all 22 biomarkers., Briefly, PCA enables reduction of a larger number of variables into a smaller number of summary components, while still retaining as much of the variance in the original variables as possible. Prior to PCA, the biomarkers were log-transformed (for approximate normality) and scaled to ensure biomarkers with larger intrinsic variation did not dominate the PCA. Unsupervised hierarchical clustering was then performed on the principal components (PCs), using Ward’s minimum variance method and squared Euclidean distance as the distance measure, and determining the optimal number of clusters using the Silhouette method [35]. For PCA, we used a single imputation for absolute values of missing biomarker data (ranging from 0.5% to 20%) for patients with any biomarker values using the SVDImpute algorithm (which imputes based on eigenvectors, analogous to PCA) within the R impute package [36].

We used logistic regression to investigate predictors of CMV positivity, adjusting for clinical risk factors, using multivariable fractional polynomials to account for nonlinear effects of biomarkers. We then examined the association between changes in cfPWV (log_10_-transformed for normality) over time and inflammatory clusters using linear mixed models with patient-specific random intercepts. We adjusted the model for baseline cfPWV values, confounders (age and sex), and mediators (blood pressure and hemoglobin), as previously identified [3].

Analysis was undertaken using Stata 13.1 (StataCorp, College Station, TX) and R V3.5.1 (The R Foundation for Statistical Computing, Vienna, Austria).

## RESULTS

In total, 279 participants positive for HIV were enrolled. The median age was 36 years (interquartile range [IQR], 31–43 years), nadir CD4 count was 41 cells/µL (18–62 cells/µL), and HIV viral load was 110 000 copies/mL (4000–290 000 copies/mL). The median (IQR) blood pressure was 120/73 mm Hg (108/68–128/80 mm Hg) and total cholesterol was 3.6 mmol/L (3.0–4.4 mmol/L). A total of 110 participants negative for HIV had comparable age and blood pressure (median [IQR], 35 [31–41] years and 128/75 [114/68–134/82] mm Hg). All but 1 patient with HIV received first-line ART of tenofovir-lamivudine-efavirenz, with the remaining participants receiving zidovudine-lamivudine-nevirapine. Forty-five (16%) participants with HIV were diagnosed with an acute coinfection at study enrollment, including 30 with TB, 4 with cryptococcal meningitis, and 4 with malaria. CMV PCR was positive for 61 (32%) of 193 tested participants with HIV and 1 (2%) of 59 participants negative for HIV at enrollment, with median (IQR) CMV viral load of 928 copies/mL (412–3052 copies/mL) in those who were positive. CMV PCR became undetectable for all except 1 participant positive for HIV after 42 weeks of ART. The median (IQR) cfPWV for the participants positive for HIV was 7.3 m/s (6.5–8.2 m/s).

Fifteen participants died. Of 14 PLWH who died, 5 deaths were due to TB, 3 to cryptococcal meningitis, 1 to pneumonia, 1 to Kaposi sarcoma, 1 to gastroenteritis, and 3 of unknown cause. One participant negative for HIV died from a cerebrovascular accident on a background of hypertension and obesity.

### Inflammatory Biomarkers

Inflammatory biomarker data were available for 211 (76%) participants positive for HIV and 62 (56%) participants negative for HIV. At enrollment, almost all markers were significantly elevated in those with HIV compared with those without, particularly anti-inflammatory cytokines (IL-10 and IL-1Ra: 2.9- and 1.9-fold higher), T-cell activation (IFN-γ and IL-7: 2.3- and 4.9-fold higher), and angiogenesis (bFGF and VEGF: 4.9- and 2.6-fold higher) (Supplementary Table 1). All inflammatory biomarkers except for IL-13 decreased in participants with HIV during the following 42 weeks on ART. However, levels of TNF-α, IL-7, MCP-1, IP-10, VEGF, bFGF, ICAM, and CRP remained significantly elevated compared with patients without HIV at the final study visit (P = .001) (Supplementary Table 2).

While many cytokines were univariably associated with CMV positivity, only associations with VCAM and MIP-1β, and baseline CD4, persisted in multivariable models after adjustment for age and sex (*P* = .04 and 0.01, respectively) (Supplementary Table 3).

Among participants with HIV, individual biomarkers were not associated with cfPWV at enrollment on univariate analysis (*P* > .05), with the exception of IL-13 (Spearman ρ, −0.21; *P* = .01) (Supplementary Table 4).

### Characterization of Immune Phenotypes During Antiretroviral Therapy Initiation

**Figure 1.**
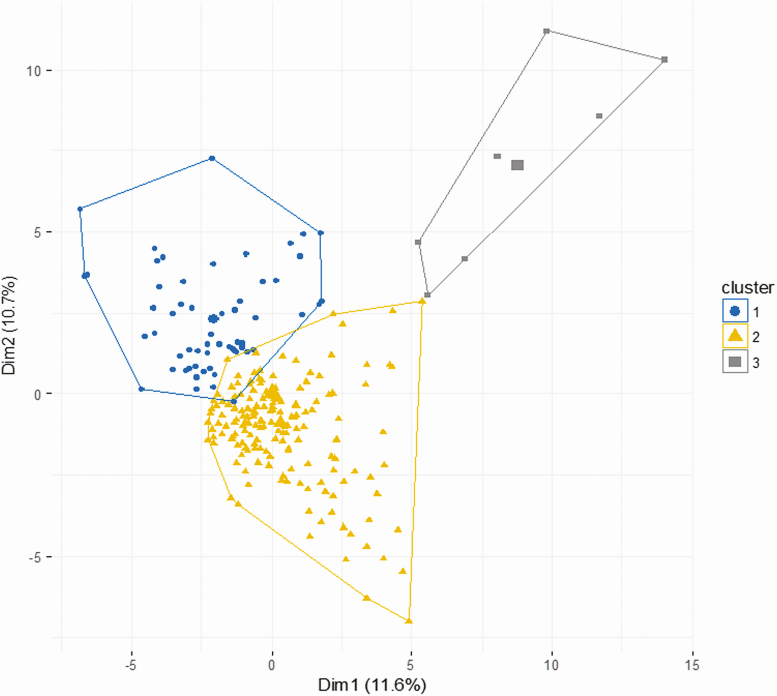
Identification of 3 distinct inflammatory biomarker clusters for 211 patients with HIV infection. Clusters plotted against the first 2 principal components (percentage total variation explained). Abbreviations: Dim, dimension; HIV, human immunodeficiency virus.

**Figure 2.**
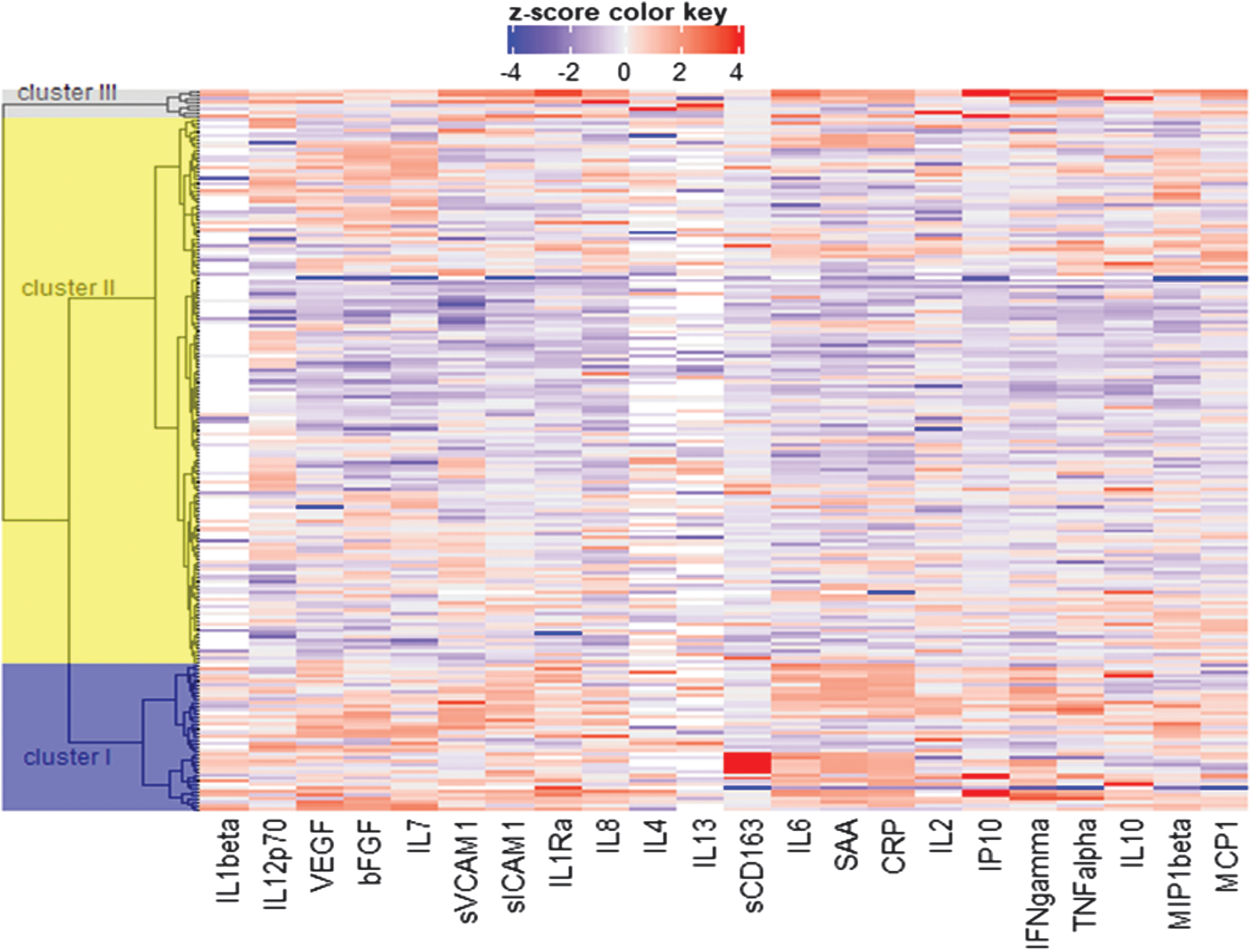
Heat map of 22 inflammatory biomarkers according to inflammatory cluster. Abbreviations: bFGF, basic fibroblastic growth factor; CRP, C-reactive protein; IL, interleukin; IFN, interferon; IP, interferon gamma-induced protein; MCP, monocyte chemoattractant protein; MIP, macrophage inflammatory protein; sVCAM, soluble vascular cell adhesion molecule; sICAM, intracellular adhesion molecule; sCD, soluble cluster of differentiation; SAA, serum amyloid A; TNF, tumor necrosis factor; VEGF, vascular endothelial growth factor.

Unsupervised hierarchical clustering of the 22 inflammatory biomarkers was performed for the 211 participants with HIV with any biomarker data. Three clusters were identified representing 51 (24%) (cluster 1), 153 (73%) (cluster 2), and 7 (3%) (cluster 3) participants (Figure 1). Figure 2 and Supplementary Table 5 compare the inflammatory biomarkers across clusters.

Although cluster 3 was small, participants had markedly increased proinflammatory cytokines compared with cluster 1 and cluster 2. Concentrations of IL-6, CRP, and IFN-γ were between 10- and 50-fold higher (all *P* < .0001) (Supplementary Table 5) and levels of serum amyloid were over 100-fold higher (*P* = .0003). For participants in cluster 1, CRP and SAA were increased compared with cluster 2 (both *P* = .02), but to a lesser extent than the levels seen in cluster 3 (Supplementary Table 5).

Table 1 shows clinical characteristics and cell-surface immune activation markers of patients in each cluster, and Supplementary Table 6 shows the relevant characteristics for those without available biomarker data. There was no evidence of differences in age, blood pressure, diagnosis of acute coinfection, or CMV positivity between clusters (*P* = .77, .47, .72, and .90, respectively). There was a trend towards lower nadir CD4 count in cluster 3 compared with cluster 2 (CD4, 17 cells/µL [11–44] vs 41 cells/µL [19–63]; *P* = .08), but no evidence of overall difference across the 3 groups (*P* = .44).

**Table 1.** Clinical Characteristics of Inflammatory Clusters

	Cluster 1 (n = 51)		Cluster 2 (n = 153)		Cluster 3 (n = 7)		*P*
	Median or Frequency	IQR or %	Median or Frequency	IQR or %	Median or Frequency	IQR or %	
Clinical characteristics							
Age, y	38	31–44	36	31–43	40	31–43	.77
Systolic BP, mm Hg	124	109–132	119	108–128	128	106–129	.47
Diastolic BP, mm Hg	73	68–78	72	68–80	78	72–89	.26
Heart rate, bpm	80	72–99	82	72–98	74	64–87	.34
Temperature, °C	36.4	36.0–36.8	36.4	36.0–36.9	36.5	36.0–36.9	.88
Hemoglobin, g/dL	11.5	10.1–12.6	11.4	9.9–13.0	12.7	11.9–13.0	.51
Creatinine, µmol/L	62	52–72	66	56–79	60	51–65	.029
Platelets, ×10^9^/L	215	172–325	240	189–332	216	144–435	.34
Nadir CD4, cells/mm^3^	42	19–65	41	19–62	17	11–44	.44
HIV viral load, copies/mL	117 000	46 000–249 000	111 000	39 000–295 000	210 000	86 000–601 000	.51
Acute coinfection, %	11	22	45	29	1	14	.72
CMV PCR positive, %	16	33	43	28	2	40	.90
Death, %	0	0	14	9	0	0	.097
Arterial stiffness							
Week 2 cfPWV, m/s	7.5	7.4–7.6	7.3	7.2–7.4	6.6	6.3–6.8	.041
Week 12 cfPWV, m/s	7.4	6.9–7.6	7.3	6.5–7.6	6.9	6.5–7.5	.30
Week 24 cfPWV, m/s	7.3	6.5–7.5	7.1	6.5–7.4	6.8	6.3–7.5	.76
Week 44 cfPWV, m/s	7.4	7.0–7.9	7.1	7.0–7.9	7.5	6.6–8.1	.75
Immune cell surface phenotype							
%CD4 activation	86	76–90	68	54–76	69	50–96	<.0001
%CD4 exhaustion	69	63–76	39	25–52	33	17–49	<.0001
%CD4 senescence	18	9–29	14	8–22	17	17–18	.42
%CD8 activation	84	76–90	72	59–86	83	65–96	<.0001
%CD8 exhaustion	54	44–61	33	23–42	42	37–48	<.0001
%CD8 senescence	51	43–59	54	44–66	54	51–63	.45
%Intermediate monocytes	7.7	4.6–11.9	10.3	6.7–13.8	12.8	9.8–14.9	.004

Abbreviations: BP, blood pressure; bpm, beats per minute; cfPWV, carotid femoral pulse-wave velocity; CMV, cytomegalovirus; HIV, human immunodeficiency virus; IQR, interquartile range; PCR, polymerase chain reaction.

Participants in cluster 1 were characterized by higher proportions of PD-1 expressing CD4 and CD8 T cells compared with cluster 2 and cluster 3 (Table 1) (CD4: median, 69% vs 39% and 33%, respectively; CD8: 54% vs 33% and 42%, respectively; both *P* < .0001) as well as a lower proportion of intermediate monocytes (7.7 vs 10.3 vs 12.8, respectively; *P* = .004). Lower proportions of activated CD4 and CD8 T cells were found in cluster 2 (CD4: 68% vs 86% in cluster 1 and 69% in cluster 3; CD8: 72% vs 84% vs 83%, respectively; *P* < .0001). However, all 14 deaths in participants positive for HIV were in cluster 2 (*P* = .097).

### Relationship Between Immune Phenotype at Antiretroviral Therapy Initiation and Arterial Stiffness

Median (IQR) cfPWV at baseline for clusters 1, 2, and 3 was 7.5 m/s (7.4–7.6), 7.3 m/s (7.2–7.4), and 6.6 m/s (6.3–6.8), respectively (*P* = .04). When adjusted for a priori–identified confounders and mediators in multivariate analysis, baseline cfPWV was lower in cluster 2 and cluster 3 compared with cluster 1 [cluster 2: fold-change, −9% (95% confidence interval [CI], −17% to 1%), *P* = .08; cluster 3: fold-change, −17% (95% CI, −53% to −15%), *P* = .002] (Table 2]. The effect of inflammatory cluster on baseline cfPWV was not attenuated when CMV positivity was added to this model (cluster 2: fold-change, −10% [95% CI, −19% to 0%], *P* = .04; cluster 3: fold-change, −37% [95% CI, −56% to −11%], *P* = .008).

**Table 2.** Linear Regression Model Showing the Adjusted Association Between Inflammatory Cluster and Arterial Stiffness at 2 Weeks Post–Antiretroviral Therapy Initiation

	Fold-change in cfPWV at Baseline, m/s	95% Confidence Interval	*P* Value
Cluster 2^a^	0.91	.83–1.01	.08
Cluster 3^a^	0.63	.47–0.85	.002
Age (years) (per 10 years older)	1.18	1.12–1.23	<.0001
Female sex	1.00	.91–1.10	.96
Systolic BP (mm Hg) (per 10 mm Hg higher)	1.03	1.00–1.06	.07
Diastolic BP (mm Hg) (per 10 mm Hg higher)	1.09	1.03–1.15	.002
Hemoglobin (g/dL) (per g/dL higher)	1.01	.99–1.04	.23

Abbreviations: BP, blood pressure; cfPWV, carotid femoral pulse-wave velocity.

^a^In comparison to cluster 1.

**Figure 3.**
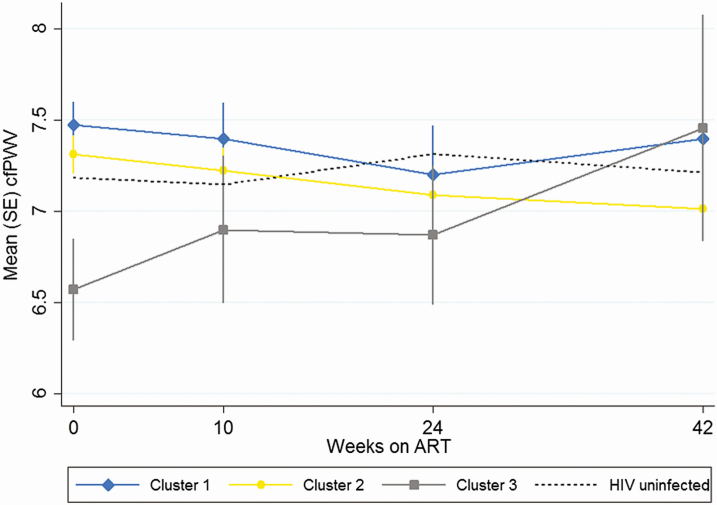
Change in cfPWV over 42 weeks of ART according to inflammatory cluster. Abbreviations: ART, antiretroviral therapy; cfPWV, carotid femoral pulse-wave velocity; HIV, human immunodeficiency virus.

Between week 2 and week 42, change in cfPWV for clusters 1, 2, and 3 was −0.5 m/s (95% CI, −1.0 to +.1), −0.4 m/s (95% CI, −1.0 to +.2), and + 0.1 m/s (95% CI, −.6 to + 1.7), respectively (Figure 3). Mixed-effects models were constructed to assess the effect of inflammatory biomarker cluster on cfPWV slope over 42 weeks when adjusted for a priori–identified confounders and mediators (Table 3). Compared with the rate of change in cluster 1, fold-change for cluster 2 was 0.99 (95% CI, .86–1.14; *P* = .91) and for cluster 3 was 1.45 (95% CI, 1.01–2.09; *P* = .045).

**Table 3.** Mixed-Model Analysis Showing the Effect of Inflammatory Biomarker Cluster on Carotid Femoral Pulse-wave Velocity Slope Over 42 Weeks Post–Antiretroviral Therapy Initiation

	Fold-change in cfPWV	95% Confidence Interval	*P* value
Average effect of time on cfPWV at 42 weeks post-ART	0.94	.90–.98	.007
Average effect of cluster on cfPWV at 42 weeks post-ART			
Cluster 2 versus cluster 1	0.95	.85–1.05	.30
Cluster 3 versus cluster 1	0.89	.69–1.14	.35
Adjusted effect of cluster on rate of change in cfPWV over 42 weeks of ART^a^			
Cluster 2 versus cluster 1	0.99	.86–1.14	.91
Cluster 3 versus cluster 1	1.45	1.01–2.09	.045
Enrollment age (per 10 years older)	1.23	1.18–1.27	<.0001
Enrollment diastolic BP (per 10 mm Hg higher)	1.10	1.06–1.15	<.0001
Female sex (vs male)	0.97	.90–1.04	.36
Enrollment hemoglobin (g/dL) (per g/dL higher)	1.01	1.00–1.03	.11

Note: Adjusted for baseline cfPWV.

Abbreviations: ART, antiretroviral therapy; BP, blood pressure; cfPWV, carotid femoral pulse-wave velocity.

^a^Model showing effect of interaction between cluster and time on cfPWV over 42 weeks post–ART initiation, adjusted for average effect of age, BP, sex, and hemoglobin.

## DISCUSSION

Patients living with HIV infection and presenting with advanced immune suppression can be classified according to distinct inflammatory phenotypes. We identified 2 inflammatory phenotypes: one characterized by T-cell activation and, in particular, high levels of PD-1 expression, and another smaller subgroup with markedly elevated inflammatory cytokines and a higher proportion of intermediate monocytes. These phenotypes tended to experience less favorable trends in arterial stiffness on ART compared with the third phenotype, which had decreased T-cell activation and decreased cfPWV on ART.

Three pathways in particular were of interest in cluster 3. Biomarkers of angiogenesis, bFGF and VEGF, have previously been linked to aberrant fibrosis, a possible mechanism for the development of noncommunicable complications on ART [37]. High IFN-γ and IL-7 levels indicate a role for lymphocyte recruitment in this cluster. This is consistent with recent data showing production of IFN-γ from cytotoxic T lymphocytes in HIV infection occurs due to delayed killing of resistant HIV-infected macrophages, which potentiate the inflammation cycle [38]. Anti-inflammatory cytokines were also upregulated in this cluster, with high levels of IL-10 and IL-1Ra. However, all deaths—which were predominantly due to infections—were in cluster 2 (the largest subgroup) where relatively lower levels of inflammation were seen. Taken together, we could hypothesize a survival benefit for those able to produce and regulate a more marked inflammatory response [39], with endothelial damage the cost in the longer term.

In contrast, cluster 1 was not defined by marked increases in inflammatory biomarkers but was associated with higher proportions of activated T cells, especially those expressing PD-1. PD-1–expressing T cells have been previously shown to be independently associated with changes in cfPWV in this cohort [3]. Given that cell-surface markers were not added to the cluster model (only the plasma biomarker data), this finding is interesting and the inflammatory biomarker signature warrants further investigation. We did not find any evidence that CMV viremia explains inflammation-related arterial stiffness in this cluster, although it was associated with plasma markers of endothelial damage overall. However, our sample size was not large enough to detect a small effect of CMV on arterial stiffness.

Few studies to date have specifically dealt with inflammation in people with CD4 of less than 100 cells/µL who are commencing ART. As well as a low CD4 count predisposing to coinfections that may trigger inflammation, the inflammatory response itself might potentiate CD4 T-cell loss. Shive and colleagues [40] previously demonstrated that proinflammatory cytokines driving CD4 T-cell cycling led to increased cell turnover and diminished responsiveness to IL-7. As more patients presenting with low CD4 counts survive past the initial few months of ART, characterizing inflammatory phenotypes at ART initiation may provide insights into the heterogeneity of chronic inflammation in the longer term [41, 42] and identify potential areas for intervention.

Previous studies from SSA have investigated the relationship between inflammation and vascular dysfunction. In a South African cohort, markers of endothelial damage including VCAM and ICAM were raised in people with HIV compared with negative controls and levels of VCAM were associated with CD4 count [43]. Siedner and colleagues [44] demonstrated that changes in sCD14 and IL-6 during the first 6 months of ART predicted a lower carotid intima thickness measurement after 7 years of ART. Decreases in CRP and TNF-α correlated with improvements in vascular function in a small cohort of malnourished patients in southern Africa. Our data add to current knowledge, suggesting that inflammatory phenotype at ART initiation may determine different trajectories of vascular function over time.

Sherzer and colleagues [45] investigated cross-sectional biomarker clustering for patients with HIV on ART in the United States, in the context of cardiac failure. Using a panel of 8 inflammatory and heart failure markers, they identified 1 clusters, one of which was also a proinflammatory phenotype and was associated with higher mortality. Banchereau and colleagues [26] used inflammatory biomarkers and transcriptomics to stratify SLE phenotypes according to underlying mechanistic pathways and identify clinically applicable biomarkers and treatments for each group.

Study strengths include plasma biomarker data in a large, clinically well-characterized cohort of patients with HIV, recruited close to ART initiation. Previous studies have concentrated on cross-sectional analysis; here, we assessed changes in plasma biomarkers, cell-surface markers, and arterial stiffness during the first 42 weeks of ART. In addition, we also included an HIV-negative cohort for comparison at both time points.

Limitations include the fact that the number of participants in cluster 3 was very small, restricting inference about their characteristics. However, these patients may represent those at highest risk of inflammation-driven complications of HIV and those most likely to benefit from targeted interventions. Although we did not find any differences that suggest this group of patients was experiencing greater rates of acute coinfection or sepsis, this remains a possibility given the low numbers. However, cfPWV remained lower in this group than the rest of the cohort even at week 10, making it unlikely that sepsis alone was an explanation for the lower arterial stiffness observed at enrollment.

Our cohort had advanced immune suppression, with CD4 of less than 100 cells/µL at ART initiation, restricting generalizability to the population with HIV as a whole. Indeed, inflammatory phenotypes may differ depending on HIV stage, as well as treatment status, and further work will be needed for those with higher CD4 counts and those established on ART. Although arterial stiffness represents one mechanism through which clinical cardiovascular disease may occur, large, clinically validated studies will also be required in the future.

Reproducible inflammatory phenotypes could inform clinical trials seeking to reverse chronic inflammation. To date, trials in this area of interventions such as statins, valganciclovir, dietary supplements, metformin, and anti-inflammatory immunomodulatory agents have shown only modest effects [46–50]. One possibility is that the effects of interventions may have been limited by the heterogeneity of the patient populations and would be enhanced when targeted towards a particular inflammatory phenotype.

This study identifies potential clinically relevant inflammatory phenotypes in PLWH. In addition, we present novel data on the possible association between inflammatory phenotypes and change in vascular dysfunction on ART. Further elucidation of the clinical implications as well as drivers of inflammatory phenotypes might help identify therapeutic targets to modulate inflammation-driven cardiovascular disease in PLWH.

## Supplementary Data

Supplementary materials are available at *Clinical Infectious Diseases* online. Consisting of data provided by the authors to benefit the reader, the posted materials are not copyedited and are the sole responsibility of the authors, so questions or comments should be addressed to the corresponding author.

ciaa186_suppl_Supplementary-Material

## Notes

***REALITY Trial Group.*** *Participating centers*: Joint Clinical Research Centre (JCRC), Kampala, Uganda (coordinating center for Uganda): P. Mugyenyi, C. Kityo, V. Musiime, P. Wavamunno, E. Nambi, P. Ocitti, M. Ndigendawani; JCRC, Fort Portal, Uganda: S. Kabahenda, M. Kemigisa, J. Acen, D. Olebo, G. Mpamize, A. Amone, D. Okweny, A. Mbonye, F. Nambaziira, A. Rweyora, M. Kangah, and V. Kabaswahili; JCRC, Gulu, Uganda: J. Abach, G. Abongomera, J. Omongin, I. Aciro, A. Philliam, B. Arach, E. Ocung, G. Amone, P. Miles, C. Adong, C. Tumsuiime, P. Kidega, B. Otto, F. Apio; JCRC, Mbale, Uganda: K. Baleeta, A. Mukuye, M. Abwola, F. Ssennono, D. Baliruno, S. Tuhirwe, R. Namisi, F. Kigongo, D. Kikyonkyo, F. Mushahara, D. Okweny, J. Tusiime, A. Musiime, A. Nankya, D. Atwongyeire, S. Sirikye, S. Mula, N. Noowe; JCRC, Mbarara, Uganda: A. Lugemwa, M. Kasozi, S. Mwebe, L. Atwine, T. Senkindu, T. Natuhurira, C. Katemba, E. Ninsiima, M. Acaku, J. Kyomuhangi, R. Ankunda, D. Tukwasibwe, L. Ayesiga; University of Zimbabwe Clinical Research Centre, Harare, Zimbabwe: J. Hakim, K. Nathoo, M. Bwakura-Dangarembizi, A. Reid, E. Chidziva, T. Mhute, G. C. Tinago, J. Bhiri, S. Mudzingwa, M. Phiri, J. Steamer, R. Nhema, C. Warambwa, G. Musoro, S. Mutsai, B. Nemasango, C. Moyo, S. Chitongo, K. Rashirai, S. Vhembo, B. Mlambo, S. Nkomani, B. Ndemera, M. Willard, C. Berejena, Y. Musodza, P. Matiza, B. Mudenge, V. Guti; KEMRI Wellcome Trust Research Program, Kilifi, Kenya: A. Etyang, C. Agutu, J. Berkley, K. Maitland, P. Njuguna, S. Mwaringa, T. Etyang, K. Awuondo, S. Wale, J. Shangala, J. Kithunga, S. Mwarumba, S. Said Maitha, R. Mutai, M. Lozi Lewa, G. Mwambingu, A. Mwanzu, C. Kalama, H. Latham, J. Shikuku, A. Fondo, A. Njogu, C. Khadenge, B. Mwakisha; Moi University Clinical Research Centre, Eldoret, Kenya: A. Siika, K. Wools-Kaloustian, W. Nyandiko, P. Cheruiyot, A. Sudoi, S. Wachira, B. Meli, M. Karoney, A. Nzioka, M. Tanui, M. Mokaya, W. Ekiru, C. Mboya, D. Mwimali, C. Mengich, J. Choge, W. Injera, .K Njenga, S. Cherutich, M. Anyango Orido, G. Omondi Lwande, P. Rutto, A. Mudogo, I. Kutto, A. Shali, L. Jaika, H. Jerotich, M. Pierre; Department of Medicine and Malawi-Liverpool Wellcome Trust Clinical Research Program, College of Medicine, Blantyre, Malawi: J. Mallewa, S. Kaunda, J. Van Oosterhout, B. O’Hare, R. Heyderman, C. Gonzalez, N. Dzabala, C. Kelly, B. Denis, G. Selemani, L. Nyondo Mipando, E. Chirwa, P. Banda, L. Mvula, H. Msuku, M. Ziwoya, Y. Manda, S. Nicholas, C. Masesa, T. Mwalukomo, L. Makhaza, I. Sheha, J. Bwanali, M. Limbuni; Trial Coordination and Oversight: MRC Clinical Trials Unit at University College London, London, United Kingdom: D. Gibb, M. Thomason, A. S. Walker, S. Pett, A. Szubert, A. Griffiths, H. Wilkes, C. Rajapakse, M. Spyer, A. Prendergast, N. Klein; Data Management Systems: M. Rauchenberger, N. Van Looy, E. Little, K. Fairbrother; Social Science Group: F. Cowan, J. Seeley, S. Bernays, R. Kawuma, Z. Mupambireyi.

***Independent REALITY Trial Monitors.*** F. Kyomuhendo, S. Nakalanzi, J. Peshu, S. Ndaa, J. Chabuka, N. Mkandawire, L. Matandika, C. Kapuya; Trial Steering Committee: I. Weller (Chair), E. Malianga, C. Mwansambo, F. Miiro, P. Elyanu, E. Bukusi, E. Katabira, O. Mugurungi, D. Gibb, J. Hakim, A. Etyang, P. Mugyenyi, J. Mallewa; Data Monitoring Committee: T. Peto (Chair), P. Musoke, J. Matenga, S. Phiri.

***Endpoint Review Committee (Independent Members).*** H. Lyall (Co-Chair), V. Johnston (Co-Chair), F. Fitzgerald, F. Post, F. Ssali, A. Prendergast, A. Arenas-Pinto, A. Turkova, A. Bamford.

***Acknowledgments.*** The authors acknowledge the patients and their families as well as the staff in the ART clinic and Department of Medicine at Queen Elizabeth Central Hospital, Blantyre, Malawi. We thank the REALITY trial group for their support with study design and implementation.

***Financial support.*** This work was supported by a Wellcome Trust Clinical PhD Training Fellowship (grant number 099934/Z/12/A), a strategic award from the Wellcome Trust for the MLW Clinical Research Programme and core support to the MRC Clinical Trials Unit at UCL (grant numbers MC_UU_12023/23 and MC_UU_12023/26) and Joint Global Health Trials (UK Medical Research Council, Wellcome Trust, Department for International Development). The Meso Scale Discovery assays were funded by Merck, Sharp, and Dohme. Cipla Ltd, Gilead Sciences, ViiV Healthcare/GlaxoSmithKline, and Merck, Sharp, and Dohme donated drugs for REALITY. A. S. W. is an NIHR Senior Investigator and reports money paid to her institution from Gilead Sciences, and personal fees from Janssen outside the submitted work. W. T. has received support from the European Union’s Horizon 2020 Research and Innovation Programme under the Marie Sklodowska-Curie (grant number 666010). S. K. reports grants and personal fees from ViiV Healthcare, grants from Gilead Sciences, and grants from Merck, outside the submitted work.

***Potential conflicts of interest.*** The authors: No reported conflicts of interest. All authors have submitted the ICMJE Form for Disclosure of Potential Conflicts of Interest.
