# Supplementary material for: Inflammatory Phenotypes Predict Changes in Arterial Stiffness Following Antiretroviral Therapy Initiation
Source: Clin Infect Dis. 2020 Jul 2;71(9):2389–97. doi: 10.1093/cid/ciaa186 (PMC7713681; doi:10.1093/cid/ciaa186)
Supplement: ciaa186_suppl_Supplementary-Material [file ciaa186_suppl_supplementary-material.docx]

**Supplementary Table 1. Concentrations of 22 inflammatory biomarkers according to HIV status**

| **Pathway** | **Marker**  **(pg/mL)^a^** | **HIV negtive (n=62)** | | | **HIV positive (n=211)** | | | **p value** |
| --- | --- | --- | --- | --- | --- | --- | --- | --- |
|  |  | **Median** | **95% Confidence Intervals** | | **Median** | **95% Confidence Intervals** | |  |
| **Pro-inflammatory cytokines** | IL6 | 0.56 | 0.34 | 1.13 | 1.44 | 0.78 | 3.90 | <0.0001 |
|  | IL1β | 0.13 | 0.05 | 1.10 | 0.30 | 0.10 | 0.70 | 0.80 |
|  | TNFα | 3.1 | 2.5 | 3.5 | 7.2 | 5.3 | 10.6 | <0.0001 |
| **Anti-inflammatory** | IL10 | 0.45 | 0.31 | 0.72 | 1.31 | 0.75 | 2.38 | <0.0001 |
|  | IL1Ra | 166 | 124 | 255 | 319 | 213 | 603 | <0.0001 |
| **T cell activation** | IL 12p70 | 0.17 | 0.10 | 0.25 | 0.23 | 0.15 | 0.35 | 0.0098 |
|  | IL2 | 0.27 | 0.15 | 0.57 | 0.57 | 0.42 | 0.87 | <0.0001 |
|  | IFNɣ | 2.7 | 1.7 | 5.1 | 12.2 | 6.1 | 26.3 | <0.0001 |
|  | IL7 | 2.4 | 1.7 | 4.7 | 11.7 | 6.8 | 20.3 | <0.0001 |
|  | IL4 | 0.04 | 0.02 | 0.07 | 0.03 | 0.02 | 0.05 | 0.53 |
| **B cell activation** | IL13 | 1.4 | 0.7 | 2.5 | 1.0 | 0.6 | 1.5 | 0.0113 |
| **Chemotaxis** | IL8 | 2.5 | 1.6 | 5.5 | 5.9 | 2.9 | 12.2 | <0.0001 |
|  | MCP1 | 54 | 45 | 65 | 86 | 66 | 114 | <0.0001 |
|  | IP10 | 202 | 165 | 311 | 870 | 523 | 1607 | <0.0001 |
|  | MIP1β | 52 | 40 | 78 | 84 | 57 | 122 | <0.0001 |
| **Innate immunity** | CD163**^a^** | 0.48 | 0.27 | 0.54 | 0.82 | 0.57 | 1.1 | <0.0001 |
| **Angiogenesis** | VEGF | 46 | 26 | 84 | 119 | 64 | 278 | <0.0001 |
|  | bFGF | 4.0 | 1.7 | 11.2 | 19.4 | 7.3 | 49.4 | <0.0001 |
| **Vascular inflammation** | ICAM**^a^** | 0.38 | 0.34 | 0.44 | 0.63 | 0.52 | 0.83 | <0.0001 |
|  | VCAM**^a^** | 0.39 | 0.33 | 0.49 | 0.58 | 0.48 | 0.72 | <0.0001 |
| **Systemic inflammation** | CRP**^a^** | 1.0 | 0.3 | 4.1 | 5.8 | 1.7 | 29.1 | <0.0001 |
|  | SAA**^a^** | 1.4 | 0.5 | 5.2 | 3.1 | 0.9 | 45.7 | 0.0005 |

**^a^**All markers are measured in pg/mL apart from those denoted with **^a^**which are in µg/mL. Note: p-values from ranksum tests.

**Supplementary Table 2. Concentrations of 22 inflammatory biomarkers according to HIV status 42 weeks post ART initiation**

| **Pathway** | **Marker** | **HIV negative** | | | **HIV positive** | | | **p value** |
| --- | --- | --- | --- | --- | --- | --- | --- | --- |
|  |  | **Median (pg/mL)^a^** | **95% Confidence Intervals** | | **Median (pg/mL)^a^** | **95% Confidence Intervals** | |  |
| **Pro-inflammatory cytokines** | IL6 | 0.59 | 0.33 | 0.97 | 0.70 | 0.43 | 1.21 | 0.02 |
|  | IL1β | 0.25 | 0.10 | 1.08 | 0.22 | 0.07 | 0.67 | 0.37 |
|  | TNFα | 3.0 | 2.6 | 3.7 | 3.8 | 3.0 | 5.0 | <0.0001 |
| **Anti-inflammatory** | IL10 | 0.43 | 0.29 | 0.67 | 0.35 | 0.25 | 0.60 | 0.049 |
|  | IL1Ra | 188 | 148 | 335 | 214 | 158 | 413 | 0.14 |
| **T cell activation** | IL 12p70 | 0.15 | 0.09 | 0.19 | 0.14 | 0.08 | 0.24 | 0.77 |
|  | IL2 | 0.21 | 0.14 | 0.38 | 0.30 | 0.19 | 0.49 | 0.0017 |
|  | IFNɣ | 3.7 | 2.0 | 4.9 | 4.0 | 2.2 | 9.6 | 0.0325 |
|  | IL7 | 2.6 | 1.6 | 5.3 | 5.0 | 2.9 | 10.0 | <0.0001 |
|  | IL4 | 0.0 | 0.01 | 0.06 | 0.02 | 0.01 | 0.05 | 0.88 |
| **B cell activation** | IL13 | 1.89 | 1.2 | 2.9 | 1.5 | 0.7 | 2.3 | 0.04 |
| **Chemotaxis** | IL8 | 3.07 | 1.6 | 6.9 | 3.3 | 1.8 | 7.2 | 0.71 |
|  | MCP1 | 56 | 40 | 69 | 69 | 52 | 94 | <0.0001 |
|  | IP10 | 222 | 151 | 306 | 359 | 280 | 584 | <0.0001 |
|  | MIP1β | 57 | 43 | 77 | 69 | 49 | 94 | 0.0052 |
| **Innate immunity** | CD163**^a^** | 0.48 | 0.36 | 0.71 | 0.55 | 0.41 | 0.76 | 0.12 |
| **Angiogenesis** | VEGF | 46 | 32 | 79 | 70 | 43 | 131 | 0.0001 |
|  | βFGF | 4 | 2 | 11 | 9 | 4 | 21 | 0.0001 |
| **Vascular inflammation** | ICAM**^a^** | 0.42 | 0.35 | 0.49 | 0.47 | 0.39 | 0.62 | 0.0007 |
|  | VCAM**^a^** | 0.44 | 0.37 | 0.53 | 0.48 | 0.39 | 0.61 | 0.0395 |
| **Systemic inflammation** | CRP**^a^** | 0.94 | 0.44 | 4.4 | 2.6 | 0.83 | 7.9 | 0.0002 |
|  | SAA**^a^** | 1.1 | 0.43 | 2.9 | 0.88 | 0.41 | 2.8 | 0.46 |

**^a^**All markers are measured in pg/mL apart from those denoted which are in µg/mL. Note: p-values from ranksum tests.

**Supplementary Table 3. Risk factors for positive CMV PCR at baseline visit in 193 participants with HIV infection**

| **Baseline factor** | **Univariate** | | | | | **Clinical Risk Factors^b^** | | | | **Clinical risk factors and inflammatory biomarkers^c^** | | | |
| --- | --- | --- | --- | --- | --- | --- | --- | --- | --- | --- | --- | --- | --- |
|  | **CMV positive** | | **CMV negative** | | **p value** |  |  |  |  |  |  |  |  |
|  | **Median or frequency** | **IQR or %** | **Median or frequency** | **IQR or %** |  | **OR** | **95 % CI min** | **95 % CI max** | **p value** | **OR** | **95 % CI min** | **95 % CI max** | **p value** |
| **Age** years | 37 | 31 – 41 | 36 | 29 – 43 | 0.89 | 1.00 | 0.94 | 1.06 | 0.96 | 0.99 | 0.94 | 1.04 | 0.62 |
| **Female sex** | 33 | 53% | 96 | 51% | 0.71 | 1.60 | 0.60 | 4.27 | 0.34 | 2.17 | 0.95 | 4.98 | 0.07 |
| **Diastolic BP** mmHg | 72 | 68 – 80 | 72 | 68 – 78 | 0.87 | 1.01 | 0.95 | 1.06 | 0.77 | - | - | - | - |
| **Haemoglobin** g/dL | 10.8 | 10 – 13 | 12 | 10 – 13 | 0.03 | 0.90 | 0.69 | 1.16 | 0.41 | - | - | - | - |
| **BMI** (kg/m^2^) | 19.2 | 17.8 – 20.8 | 19.8 | 18.6 – 21.6 | 0.03 | 0.92 | 0.79 | 1.07 | 0.29 | - | - | - | - |
| **CD4** **count** (cells/µL) | 32 | 17 – 53 | 49 | 21 – 67 | 0.03 | 0.98 | 0.96 | 1.00 | 0.03 | 0.94 | 0.90 | 0.99 | 0.008 |
| **HIV viral load** (log copies/mL) | 11.9 | 11.3 – 12.8 | 11.5 | 10.4 – 12.4 | 0.009 | 0.98 | 0.38 | 2.57 | 0.95 | - | - | - | - |
| **Other acute co-infection** | 15 | 25% | 27 | 14% | 0.06 | 0.79 | 0.26 | 2.36 | 0.67 | - | - | - | - |
| **%Activated CD8+T cells** | 82 | 71 – 92 | 75 | 62 – 85 | 0.008 | 1.03 | 1.00 | 1.06 | 0.07 | 1.04 | 1.01 | 1.07 | 0.008 |
| **Inflammatory biomarkers (pg/mL**^a^**)** | | | | | | | | | | | | | |
| IL1Ra | 381 | 270 - 682 | 286 | 187 – 473 | 0.003 | - | - | - | - | - | - | - | - |
| VCAM^a^ | 13.33 | 13.18 – 13.60 | 13.22 | 13.00 – 13.43 | 0.002 | - | - | - | - | 5.26 | 1.08 | 25.68 | 0.04 |
| ICAM^a^ | 13.46 | 13.28 – 13.78 | 13.30 | 13.08– 13.53 | 0.0002 | - | - | - | - | - | - | - | - |
| MIP1β | 107 | 70 – 137 | 72 | 52 – 109 | 0.0005 | - | - | - | - | 1.010 | 1.002 | 1.018 | 0.01 |
| IP10 | 1251 | 871 – 2374 | 772 | 487 – 1461 | <0.0001 | - | - | - | - | - | - | - | - |
| MCP1 | 102 | 75 – 125 | 78 | 63 – 110 | 0.007 | - | - | - | - | - | - | - | - |
| IL8 | 9 | 4 – 16 | 5 | 3 – 9 | 0.005 | - | - | - | - | - | - | - | - |
| IL10 | 1.8 | 1.3 – 3.4 | 1 | 0.7 – 2.0 | <0.0001 | - | - | - | - | - | - | - | - |
| TNFα | 10 | 7 – 13 | 7 | 5 – 10 | 0.0003 | - | - | - | - | - | - | - | - |

^a^All inflammatory biomarkers with a univariate association with CMV positivity (p<0.05) based on ranksum test, measured in pg/mL apart from those denoted^a^ which are in log pg/mL. **^b^**Logistic regression multivariable fractional polynomial model of all clinical factors entered simultaneously. **^c^**Logistic regression multivariable fractional polynomial model of age and sex plus those factors with significant associations found in the clinical model together with those inflammatory biomarkers found to have an adjusted association with CMV positivity, entered simultaneously; log transforms selected for VCAM.

**Supplementary Table 4. Correlation between individual biomarkers and baseline cfPWV**

| **Pathway** | Marker | Fold change**^a^** | p value |
| --- | --- | --- | --- |
| **Pro-inflammatory cytokines** | IL6 | -0.007 | 0.92 |
|  | IL1β | -0.03 | 0.79 |
|  | TNFα | -0.02 | 0.82 |
| **Anti-inflammatory** | IL10 | 0.02 | 0.79 |
|  | IL1Ra | -0.07 | 0.33 |
| **T cell activation** | IL 12p70 | -0.02 | 0.80 |
|  | IL2 | -0.05 | 0.44 |
|  | IFNɣ | -0.03 | 0.65 |
|  | IL7 | 0.05 | 0.44 |
|  | IL4 | -0.13 | 0.10 |
| **B cell activation** | IL13 | -0.21 | 0.01 |
| **Chemotaxis** | IL8 | 0.13 | 0.06 |
|  | MCP1 | 0.12 | 0.08 |
|  | IP10 | -0.02 | 0.82 |
|  | MIP1β | 0.09 | 0.19 |
| **Innate immune response** | CD163 | 0.09 | 0.21 |
| **Angiogenesis** | VEGF | 0.009 | 0.89 |
|  | βFGF | 0.11 | 0.12 |
| **Vascular inflammation** | ICAM | 0.001 | 0.99 |
|  | VCAM | 0.03 | 0.67 |
| **Systemic inflammation** | CRP | -0.04 | 0.54 |
|  | SAA | -0.03 | 0.71 |

Note: p-values from spearman rho tests. **^a^**per 1m/s increase in cfPWV

**Supplementary Table 5. Inflammatory markers included in PCA analysis and distribution across groups**

| **Pathway** | **Marker**  **(pg/mL)^a^** | **Cluster 1 (n=51)** | | **Cluster 2 (n=153)** | | **Cluster 3 (n=7)** | | **p value** |
| --- | --- | --- | --- | --- | --- | --- | --- | --- |
|  |  | **Median** | **IQR** | **Median** | **IQR** | **Median** | **IQR** |  |
| **Pro-inflammatory cytokines** | IL 6 | 1.9 | 0.8 – 5.6 | 1.2 | 0.7 – 2.3 | 19.9 | 8.6 – 39.1 | <0.0001 |
|  | IL1β | 0.43 | 0.10 – 0.76 | 0.27 | 0.11 – 0.65 | 3.21 | 1.39 – 4.82 | 0.007 |
|  | TNFα | 7.7 | 6.7 – 10.6 | 6.8 | 5.1 – 10.4 | 9.0 | 5.4 – 10.6 | 0.20 |
| **Anti-inflammatory** | IL10 | 1.1 | 0.6 – 1.8 | 1.3 | 0.8 – 2.2 | 5.0 | 2.9 – 6.8 | 0.001 |
|  | IL1RA | 314 | 174 – 592 | 304 | 206 – 420 | 2882 | 1465 – 10920 | <0.0001 |
| **T cell activation** | IL12p70 | 0.16 | 0.10 – 0.28 | 0.24 | 0.17 – 0.35 | 0.34 | 0.24 – 0.70 | 0.007 |
|  | IL2 | 0.52 | 0.31 – 0.80 | 0.58 | 0.37 – 0.86 | 0.49 | 0.44 – 0.90 | 0.46 |
|  | IFNɣ | 7 | 3 – 29 | 11 | 6 – 22 | 312 | 132 – 1087 | <0.0001 |
|  | IL7 | 12 | 6 – 18 | 12 | 7 – 20 | 21 | 15 – 22 | 0.13 |
|  | IL4 | 0.02 | 0.11 – 0.04 | 0.03 | 0.01 -0.05 | 0.03 | 0.01 – 0.09 | 0.42 |
| **B cell activation** | IL13 | 1.10 | 0.85 – 1.86 | 0.79 | 0.41 – 1.13 | 0.66 | 0.28 – 1.51 | 0.02 |
| **Chemotaxis** | IL 8 | 4.8 | 2.6 – 9.5 | 5.6 | 2.9 – 11.8 | 35.4 | 20.7 – 84.2 | 0.001 |
|  | MCP1 | 78 | 65 – 112 | 86 | 65 – 112 | 125 | 58-316 | 0.41 |
|  | IP 10 | 772 | 418 – 1621 | 846 | 501 – 1385 | 23,000 | 1500 – 230,000 | 0.002 |
|  | MIP1β | 77 | 51 – 114 | 92 | 60 – 122 | 110 | 70 – 169 | 0.14 |
| **Innate immune response** | sCD163**^a^** | 0.8 | 0.5 – 1.1 | 0.8 | 0.6 – 1.0 | 1.2 | 0.5 – 1.3 | 0.70 |
| **Angiogenesis** | VEGF | 89 | 58 – 195 | 143 | 67- 285 | 416 | 194 – 528 | 0.007 |
|  | BFGF | 20 | 11 – 40 | 26 | 7 – 65 | 27 | 21 – 38 | 0.69 |
| **Vascular inflammation** | sICAM1**^a^** | 0.6 | 0.4 – 0.9 | 0.6 | 0.5 – 0.7 | 1.9 | 1.1 – 2.2 | <0.0001 |
|  | sVCAM1**^a^** | 0.5 | 0.4 – 0.7 | 0.6 | 0.5 – 0.7 | 1.0 | 0.8 – 1.3 | 0.0004 |
| **Systemic inflammation** | CRP**^a^** | 9.9 | 2– 69 | 4 | 1 – 16 | 170 | 120 – 200 | <0.0001 |
|  | SAA**^a^** | 7 | 0.9 – 142 | 2 | 1 – 13 | 225 | 46 – 92 | 0.0003 |

**^a^** All markers are measured in pg/µL except for those denoted with **^a^**which are in µg/µL

**Supplementary Table 6: Comparison of Clinical Characteristics for Malawian adults living with HIV according to availability of biomarker data**

|  | | **Biomarker data available (n=211)** | | **Biomarker data not available (n=68)** | |
| --- | --- | --- | --- | --- | --- |
|  |  | **Median or frequency** | **IQR or %** | **Median or frequency** | **IQR or %** |
| **Clinical characteristics** | **Age (years)** | 36.3 | 30.8 – 43.0 | 37.7 | 31.8 – 44.6 |
|  | **Systolic BP (mmHg)** | 116 | 108 - 128 | 127 | 111 - 132 |
|  | **Diastolic BP** | 72 | 68 - 80 | 74 | 69 - 82 |
|  | **Heart rate (bpm)** | 82 | 72 - 100 | 78 | 72 - 92 |
|  | **Temperature (°C)** | 36.4 | 36 – 36.9 | 36.4 | 36.1 – 37.0 |
|  | **Haemoglobin (g/dL)** | 11.6 | 10.1 – 13.0 | 11.3 | 10.0 – 12.5 |
|  | **Creatinine (µmol/L)** | 64 | 54 - 77 | 68 | 58 - 82 |
|  | **Platelets (x10^9^/L)** | 236 | 185 – 334 | 223 | 189 – 304 |
|  | **Nadir CD4 (cells/mm^3^)** | 37 | 18 - 61 | 43 | 18 - 65 |
|  | **HIV viral load (copies/mL x10^6^)** | 1.1 | 0.4 – 2.8 | 1.2 | 0.4 – 2.9 |
|  | **Acute co-infection** | 43 | 20 | 14 | 22 |
|  | **CMV PCR positive** ^a^ | 60 | 28 | 1 | 2 |
|  | **Death** ^a^ | 10 | 5 | 13 | 19 |
| **Arterial stiffness** ^a^ | **Week 2 cfPWV (m/s)** | 7.3 | 6.5 – 7.9 | 8.5 | 6.6 - 10 |
|  | **Week 12 PWV (m/s)** | 7.1 | 6.5 – 7.8 | 8.1 | 6.5 – 9.3 |
|  | **Week 24 cfPWV (m/s)** | 7.0 | 6.3 – 7.7 | 7.5 | 7.0 – 8.3 |
|  | **Week 44 cfPWV (m/s)** | 6.8 | 6.3 – 7.7 | 7.6 | 6.8 – 8.4 |
| **Immune cell surface phenotype** | **%CD4 Activation** | 76 | 62 - 86 | 73 | 53 - 79 |
|  | **%CD4 Exhaustion** | 54 | 44 – 58 | 54 | 29 - 69 |
|  | **%CD4 Senescence** | 15 | 9 – 22 | 15 | 11 - 26 |
|  | **%CD8 Activation** | 79 | 65 – 88 | 75 | 59 - 86 |
|  | **%CD8 Exhaustion** | 38 | 29 – 50 | 36 | 27 - 48 |
|  | **%CD8 Senescence** | 54 | 43 – 64 | 52 | 45 - 62 |
|  | **%Intermediate Monocytes** | 9.8 | 6.0 – 13.0 | 11.5 | 6.3 – 15.0 |

^a^Consent for biomarker data analysis was obtained retrospectively towards the end of recruitment, therefore differences in CMV positivity, mortality rates and arterial stiffness may be explained by differences in those providing consent as well as loss to follow-up and death during the study. As arterial stiffness and mortality was lower amongst those included in the biomarker analysis, results described here may underestimate prevalence and cardiovascular risk for inflammatory clusters at ART initiation.
